# Chemical Variability of Essential Oils from Corsican Hops and Highlighting Their Influence on Hops’ Aroma

**DOI:** 10.3390/foods12132613

**Published:** 2023-07-06

**Authors:** Axel Dabbous-Wach, Jean-Valère Lorenzetti, Julien Paolini, Jean Costa

**Affiliations:** Unité Mixte de Recherche 6134 Sciences Pour l’Environnement, University of Corsica, Campus Grimaldi, 20250 Corte, France; lorenzetti_je@univ-corse.fr (J.-V.L.); paolini_j@univ-corse.fr (J.P.); costa_d@univ-corse.fr (J.C.)

**Keywords:** hops, aroma, essential oils, Corsica, GC–MS

## Abstract

Essential oils from wild Corsican hops have never been described before. Following selective harvesting and extraction of plant material, chemical analysis was performed by GC–FID and GC–MS. Subsequent quantitative analysis demonstrated significant inter-stations variability due to pedoclimatic conditions. These singularities produce organoleptic differences, especially within Italian hops, which are the current benchmark for the Mediterranean hops’ population. Corsican wild hops are no exception. Accordingly, three olfactive bouquets were identified by a panel of selected and trained sensory analysts: woody herbaceous ginger notes, herbaceous citrus notes, and common notes. These bouquets appeared to be correlated to pedoclimatic parameters mentioned earlier such as altitude and proximity to the sea. A very rare and appreciated bouquet was associated with high levels of zingiberene in hops growing at moderate altitude and relatively far from the coastline. This study shows the importance of growing sites and pedoclimatic conditions to produce hops with the desired organoleptic notes during the beer making process and provides detailed identification of essential oils from Corsican wild hops.

## 1. Introduction

Hops (*Humulus lupulus* L.) are one of the four components of beer. Since the 13th century, the inflorescences of these vines, called cones, have been brewed for their aroma, bitterness, and preservative properties [1]. Lupulin, a green substance stored between the bracts of the strobili, is known to contain the compounds responsible for the defensive, curative, and organoleptic characteristics of hops [1,2,3]. The chemical composition of hops’ essential oils has been well studied in the brewing industry due to their impact on the aroma profile of beers. Worldwide, several studies focused on their chemical characterization. The most common method used to identify and quantify volatile components is gas chromatography coupled with mass spectrometry, which is used in this study. The three main components identified in hops’ essential oils are usually myrcene, humulene, and caryophyllene [4,5,6,7,8,9]. These molecules and their abundance are, therefore, responsible for the development of the desired beer aroma during the process. However, the fact that components usually found in minor concentration can also contribute to the development of unusual aroma profiles (bouquets) cannot be excluded.

Hops grow spontaneously between the 35° and 55° parallel north [10]. In Corsica (43° N), wild hops are naturally present on riverbanks and mouths as well as on northern sandy soils. Even if the first mention of wild hops growing in Corsica dates back more than a century [2], their chemical profiles were only first described in one of our recently published works [11]. Moreover, the geographical variability of essential oils from spontaneous Corsican hops was evaluated for the first time in this study. It is crucial to point out that differences in essential oil content were reported for Italian hops growing over short distances due to the particularity of the Mediterranean conformation [12].

The characterization of the aroma profile of Corsican hops appears to be a priority for the expanding local brewing sector. Classification of hops’ aroma using “aroma wheels” is common in the beverage industry. Analyzing the ratio between fruity, spicy, resinous, citrus, and herbaceous notes could help brewers to determine the sensory profile of cultivars [13]. Recent studies highlight the influence of ecological and cultural parameters on the flavor profiles of hops, which have been proven to impact the aroma of beers [14,15]. These parameters, such as growing conditions, pedology, or weather, are called *terroir* and are identifiable as discriminating factors leading to drastically different organoleptic profiles [16]. To check whether these observations also apply to Corsican hops, the essential oils from thirteen stations spread over the island were analyzed over a period of three years. Subsequent statistical analyzes were conducted to determine the influence of geographical origin, pedoclimatic conditions, and year of harvesting on the chemical composition of obtained oils. The aroma profiles of the different essential oils obtained from hops growing on distinct *terroirs* were determined by blind sensory analysis with a specifically trained panel. Aspects of valorization were considered to comprehend the interest of these results on the cultural and economic development of Corsica.

The general aim of this study was to determine the geographical and temporal variabilities of essential oils from Corsican hops, depending on *terroir*, and their impact on their aroma profiles and the local brewing industry usage.

## 2. Materials and Methods

### 2.1. Plant Material

In 2019, after a 1500 km prospection around the island, cones of spontaneously growing hops were manually harvested at maturity in thirteen remote locations on Corsican riverbanks. At each station, samples were collected from three different individuals to limit possible bias due to theoretical inter individuals’ variations. This sampling protocol was repeated in 2020 and 2021 in these same locations, whose soil characteristics are indicated in Table 1. A total of 117 samples were collected. Figure A2, found in Appendix A, provides a detailed map of the 13 locations across the island.

### 2.2. Essential Oils

Essential oils from the 117 samples were prepared by hydro-distillation using a Clevenger apparatus for four hours as recommended by the European Pharmacopoeia [17], then analyzed by GC–FID and GC–MS using the following devices.

Analyzes were carried out using a PerkinElmer Autosystem GC apparatus (Whaltam, MA, USA) equipped with a dual-flame ionization detection (FID) and fused silica capillary columns (60 m × 0.22 mm i.d., film thickness 0.25 μm) with different stationary phases:  Rtx-1 (polydimethylsiloxane) and Rtx-Wax (polyethylene glycol). The oven temperature program was from 60 to 230 °C at 2 °C/min and then was held isothermally (30 min). Carrier gas was hydrogen (1 mL/min). Injector and detector temperatures were held at 280 °C. Split injection was performed with a fractional ratio of 1:80. The injected oil volume was 0.2 μL. The relative percentage of the oil constituents was calculated from the GC peak areas without using correction factors.

Mass spectrometry analyzes were conducted on a PerkinElmer TurboMass (Waltham, MA, USA) detector (quadrupole), coupled in line to a PerkinElmer Autosystem XL equipped with two fused silica capillary columns (60 m × 0.22 mm i.d., film thickness 0.25 μm), Rtx-1 (polydimethylsiloxane), and Rtx-Wax (polyethylene glycol). The other GC conditions were the same as described above. The ion source temperature was 150 °C, the energy ionization was 70 eV, and the electron ionization (EI) mass spectra were acquired over the mass range of 35−350 Da. The volume of oil injected was 0.2 μL. The main volatile compounds were identified based on their mass spectra compared with the reference mass spectra libraries (WILEY275, Wiley Science Solutions, USA; NBS75K, NIST, USA) and of their calculated retention indexes (RIs) through the application of the Kovats index (KI) formula compared with those reported in the literature and linear retention index (LRI). When it was not possible to find the KI in the literature, a tentative identification was obtained by matching with mass spectra libraries data: a match quality of 98% minimum was used as a criterion. The gas chromatographic signals were manually integrated, and the resulting peak areas were compared with the total sum of area and expressed in percentage.

For each year, the quantitative characterizations of the three samples collected at each location were averaged because of their similarity. The 39 chemical profiles obtained, and their corresponding harvesting year, are listed in Table 2.

### 2.3. Sensory Analyzes

A panel of twenty volunteers was trained to carry out sensory analyzes for several months according to the ISO 8586:2012 and 8588:2017 norms. These norms explain the selection process for effective panelists.

As requested, thirty-nine “naïve assessors” were confronted with simple blind sensory analyzes consisting of determining the presence or absence of a product with a marked fragrance (e.g., cinnamon) in a hydro-ethanolic solution contained in an opaque bottle to avoid visual bias. The twenty-eight sensitive enough panelists became “initiated assessors”. They were trained to recognize the five typical scent categories of hops (“spicy”, “woody”, “herbal”, “citrus”, and “tropical fruit”) and asked to determine their intensity from 0 to 5 within essential oils. This scale was chosen in accordance with the common range of aroma wheels used by professional brewers, and panelists were trained accordingly [18]. For these scents, the following products were used as control olfactory markers for the training process: respectively, standard mixes of (*E*)-β-caryophyllene and zingiberene (spicy), α-selinene and α-humulene (woody), β-selinene (herbal), (*E*)-β-farnesene and myrcene (citrus), and 3-mercaptohexanol (tropical fruit). These standards mixes were diluted at 5 different concentrations (20 µL·mL^−1^, 50 µL·mL^−1^, 100 µL·mL^−1^, 150 µL·mL^−1^, and 200 µL·mL^−1^) to ensure panelists exposure to different levels of aroma intensities. The higher concentrations used in this study are commonly considered to lead to strong olfactive detection for myrcene [19]. In the case of a scent expressed by two products (e.g., woody), both were diluted at equal concentrations to create the desired aroma. The range described also included a blank corresponding to theoretical zero on the defined aroma scale. In these samples on which the panelists were trained for twenty-five hours spread out weekly over six months, it was asked that the intensity of the corresponding scent be determined from 0 to 5. The different mixes at different concentrations were randomly distributed upon panelists at the beginning of each session. The panelists’ efficiency regarding repeatability was deemed satisfactory for a standard deviation of less than 1.5. Panelists unable to provide such efficiency were eliminated; the other twenty became “selected assessors”. Other exercises were conducted where panelists were given all 6 tubes from a randomly attributed scent and asked to sort them from lower to higher intensity.

At the end of the training, the twenty remaining panelists were asked to determine the intensity of the five scent categories on the thirty-nine essential oils. To avoid bias and olfactory saturation, these analyzes were triplicated and spread out over ten sessions. In these sessions, the 117 samples were randomly and blindly distributed.

### 2.4. Data Analyzes

Statistical analysis of the essential oils’ chemical compositions was performed using the open-source R studio software (version 1.2.5001, Posit Software, USA) and factoextra package (version 1.0.7). Principal component analyzes (PCAs) were carried out on different groups of data later described:

- The thirty-nine Corsican accessions (1–39) corresponding to the samples from the thirteen locations over the three years (2019, 2020, and 2021) regarding their relative abundance of the fifty-nine volatile compounds detected in the essential oils (C1–C59);
- The same thirty-nine Corsican accessions (1–39) regarding their abundance of the seven highly variable volatile compounds identified as olfactive markers: myrcene (C2), (*E*)-β-farnesene (C12), α-humulene (C13), β-selinene (C17), zingiberene (C18), and α-selinene (C20);
- The thirty-nine Corsican accessions (1–39) averaged to thirteen accessions depending on their harvest location (to avoid year-to-year variability) regarding their abundance of the fifty-nine volatile compounds detected in the essential oils (C1–C59).

Two-way ANOVAs were carried out between the ratio of the seven discriminant compounds described above and parameters such as year, proximity to the sea, and pedology. The corresponding *p*-values were considered significant when inferior to 0.05.

Additionally, the raw data corresponding to the abundance of the fifty-nine volatile compounds detected in the essential oils (C1–C59) for the thirty-nine Corsican accessions (1–39) were normalized using the centering normalization from the “scale” function in R. These data were then put in a dissimilarity matrix using Euclidean distance (“dist” function in R) and the hierarchical tree resulting from the complete matrix linkage (“hclust” function in R) was graphically plotted as a dendrogram.

## 3. Results and Discussion

### 3.1. Chemical Characterization of Essential Oils

The chemical profiles of essential oils (EO) were determined, as shown in Table A1 (given in Appendix A). Fifty-nine compounds were identified by GC–MS, which accounted for 67.4–98.4% of the EO composition. These constituents, their retention indices, and their abundances are listed in Table A2 (given in Appendix A). The volatile metabolites have been grouped into six classes based on their functions (hydrocarbons, alcohols, ketones, aldehydes, esters, and others).

**Figure A1:**
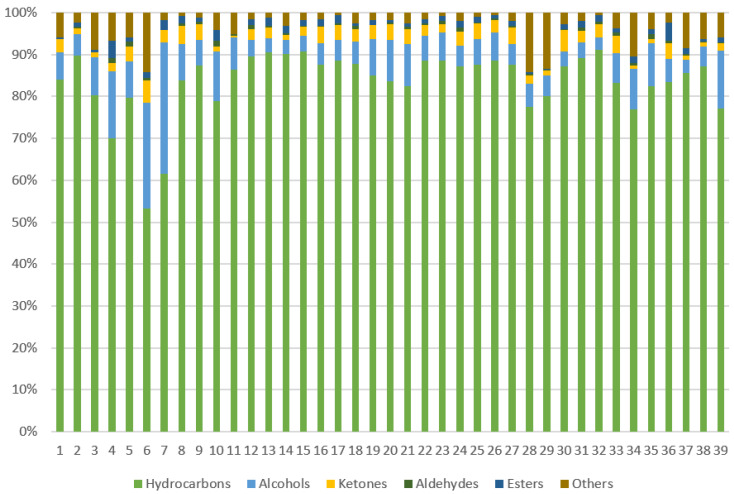
Relative composition of the 39 accessions divided into 6 compound classes.

**Figure A2:**
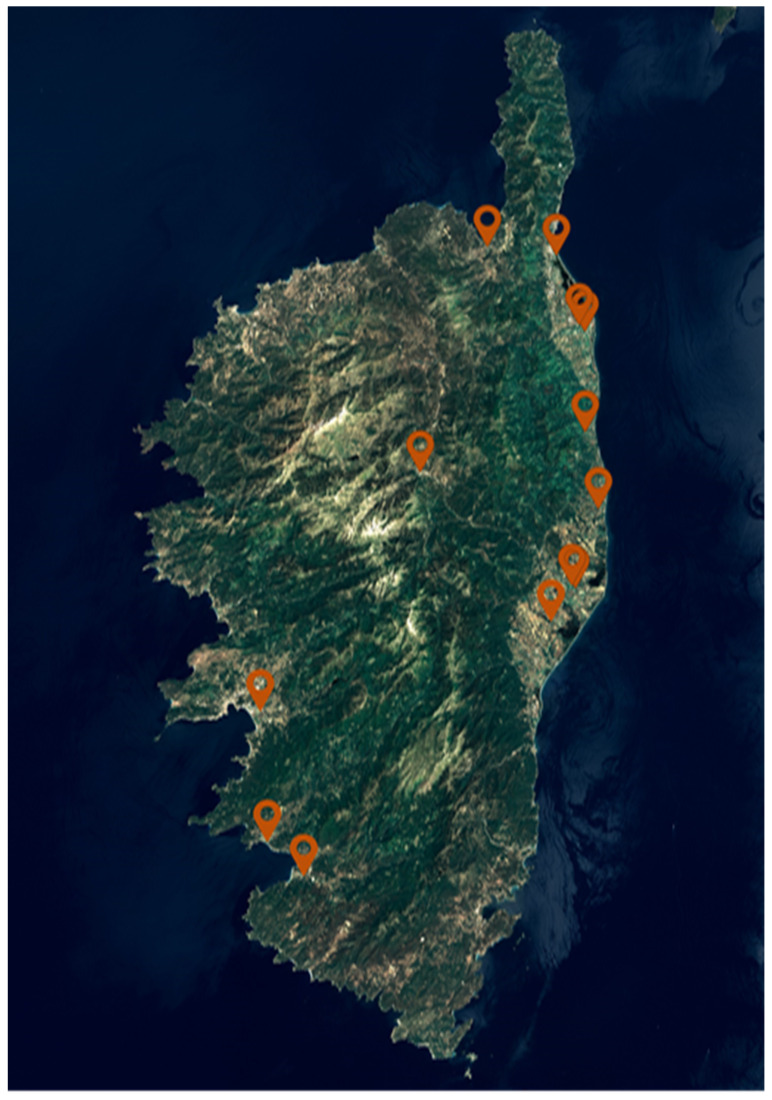
Map of harvesting sites for Corsican wild hops. From top to bottom and left to right: Oletta, Biguglia, Lucciana, Venzolasca, Santa-Maria-Poggio, Corte, Canale di Verde, Sortipiani, Casaperta, Urbino, Ajaccio, Porto-Pollo, Propriano.

The representation of the abundances of the different constituent families, represented in Figure A1 (given in Appendix A), allowed us to observe that the global contents remain similar with a few exceptions (accessions 4, 6, 7, 28, and 29). No year-related impact is observed regarding these six classes. Despite these similarities, significant variations can be observed within the hydrocarbon class, particularly for the contents of seven compounds known for their impact on hops’ aroma [20,21,22] (in *italic* in Table A1 and Table A2): C2 myrcene (*tr*—28.3%), C9 (*E*)-β-caryophyllene (0.3–30.1%), C12 (*E*)-β-farnesene (*tr*—26.6%), C13 α-humulene (0.1–29.1%), C17 β-selinene (0.5–26.6%), C18 zingiberene (*tr*—22.1%), and C20 α-selinene (*tr*—23.6%).

The relative abundances of these compounds averaged in Table 3 are different from those exposed in a recent study on Italian wild hops [23], especially for the zingiberene, which is not detected in Italian population. These differences highlight a singular volatile profile of Corsican wild accessions among Mediterranean hops. Moreover, the very high standard deviations are evidence of strong dissimilarities between the samples, which means that there is a great quantitative variability within the studied territory.

### 3.2. Statistical Analyses—Year-to-Year Variability

**Figure 1:**
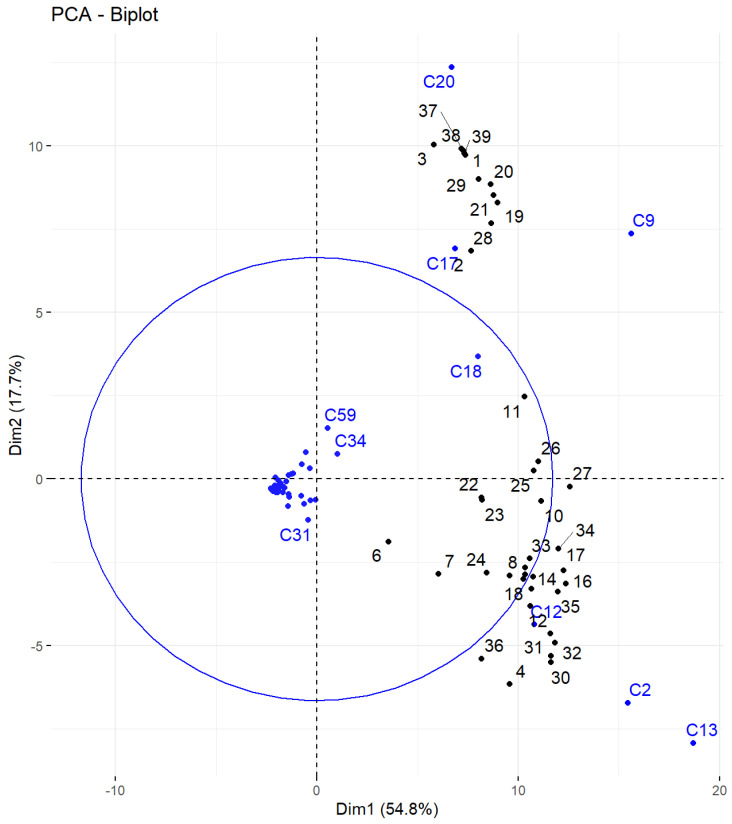
PCA of the 39 accessions (all compounds).

Chemical characterization of essential oils shows a great quantitative variability. To determine the variables that can be described as discriminant factors influencing these dissimilarities, several batches of statistical analyses were performed using the RStudio software. In the PCA performed and illustrated in Figure 1, the thirty-nine accessions (1–39) are shown in black, and the fifty-nine volatile compounds as variables (C1–C59) are shown in blue. The two dimensions selected express a total of 72.5% of total variance, which seems fairly significant considering the diversity of the parameters and the size of the data set analyzed.

**Figure 2:**
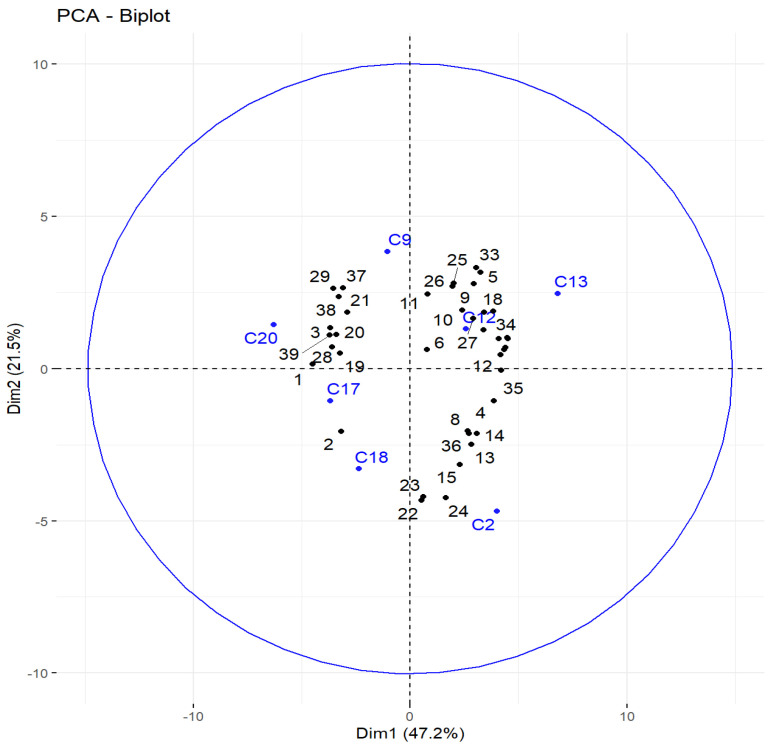
PCA of the 39 accessions (seven discriminant compounds).

A differentiation emerges between two groups of accessions according to their abundances in (*E*)-β-caryophyllene (C9), β-selinene (C17), and α-selinene (C20) for the first group, and in myrcene (C2), (*E*)-β-farnesene (C12), and α-humulene (C13) for the second. Zingiberene’s (C18) discrimination pattern is difficult to read because of the influence of other minor compounds that are not well represented by the two dimensions shown here. Therefore, another analysis was performed, focusing on the seven compounds mentioned above (Figure 2). In this figure, the thirty-nine accessions (1–39) are shown in black, and the seven volatile compounds as variables are shown in blue.

The PCA represented in Figure 2 seems to show a new differentiation pattern between four groups of accessions according to their abundance in β-selinene (C17) and α-selinene (C20) for the first group, (*E*)-β-farnesene (C12) and α-humulene (C13) for the second, myrcene (C2) for the third, and possibly zingiberene (C18) for the fourth. This last group is only composed of the accession Corte 2020 (2). These observations could depict a great chemical variability within Corsican wild hops.

This biplot also shows a great dissimilarity between some samples from the same locations but harvested over different years (e.g., Corte (1, 2, 3); Canale di Verde (4, 5, 6)) and could, therefore, highlight a temporal variability. However, it is important to mention that the significance of this PCA is not optimal considering the relatively poor representativity of the two dimensions (68.7%) expressed in this plot. Moreover, in this particular projection, the variables are relatively close to the center, indicating that their representation following these principal components is not great. Year-to-year chemical variability shows little to no clear pattern and suggests that essential oils compositions are certainly influenced by localized climatic phenomena.

### 3.3. Statistical Analyses—Geographical Diversity

**Figure 3:**
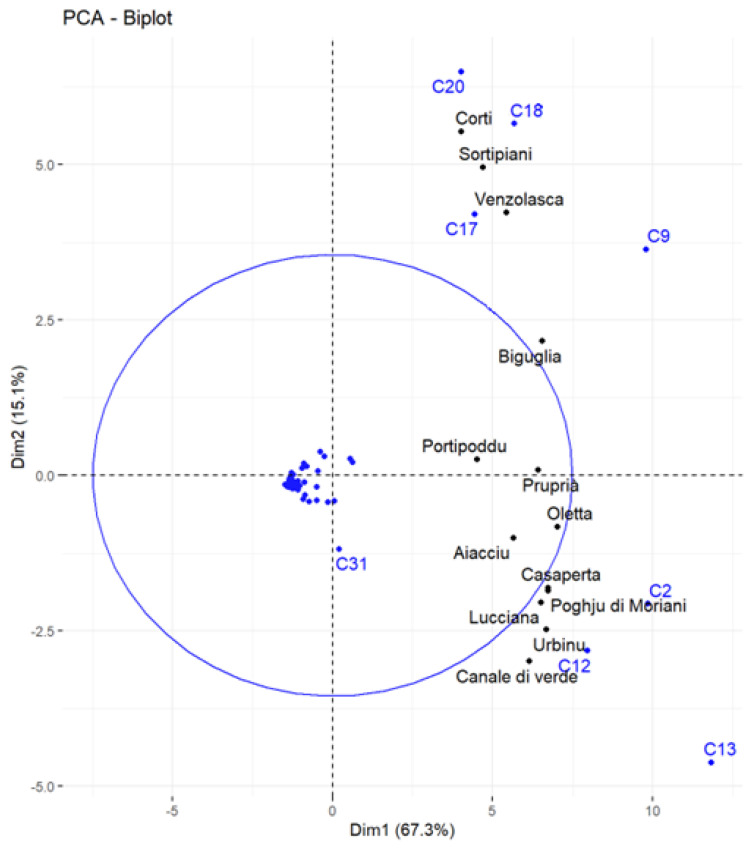
PCA of the accessions averaged by location (all compounds).

To comprehend geographical diversity, another principal components analysis was performed. Potential biases caused by year-to-year variability were limited by averaging the chemical profiles of the accessions depending on their harvesting site. The biplot obtained from the whole volatile profile is illustrated in Figure 3. In this figure, the thirteen locations are shown in black, and the fifty-nine volatile compounds as variables (C1–C59) are shown in blue.

In this representation, *Corte, Sortipiani,* and *Venzolasca* appear to show great similarity following their abundance in β-selinene (C17), zingiberene (C18), and α-selinene (C20). These three accessions appear to grow at medium altitude in the same river basin (*Tavignanu*). *Urbino, Lucciana*, and *Canale di Verde* profiles seem highly comparable depending on their abundance in (*E*)-β-farnesene (C12). To a lesser extent, *Casaperta* and *Santa-Maria-Poggio* show high similarity following their abundance in myrcene (C2). The other accessions seem equally impacted by the seven discriminant compounds. These results allow us to theorize a possible variation in olfactive bouquets of essential oils obtained from the four groups described above.

In hops, α-selinene and α-humulene are known to express woody notes, (*E*)-β-farnesene and myrcene herbal–citrus notes, β-selinene herbal notes, (*E*)-β-caryophyllene spicy notes of cloves, and zingiberene rare spicy notes of ginger [20,21,22]. Table 4 gives an estimation of the flavor notes of Corsican wild hops depending on their volatile profile and the literature [20,21,22].

As shown in Table 4, noteworthy high contents of zingiberene are only found in hops growing at medium altitude (130–400 m) away from the coast, which are areas characterized by specific pedoclimatic conditions (e.g., salinity).

**Figure 4:**
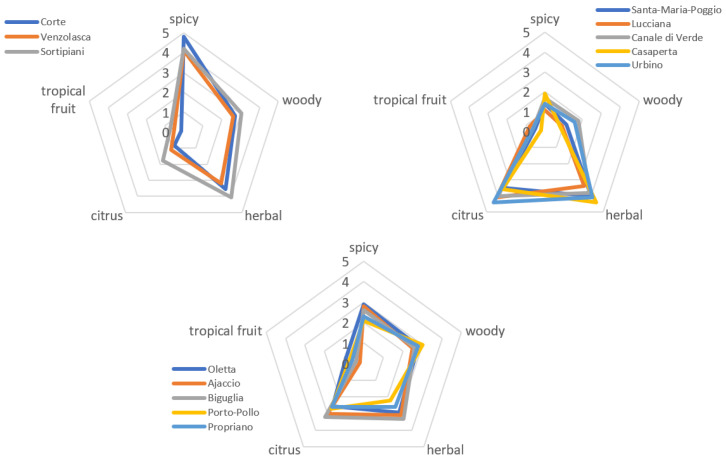
Olfactory analyzes of the EOs of the 13 accessions.

The essential oils were submitted to a trained panel for olfactory and sensory analyzes. The averaged results are shown in Figure 4. Tropical fruit notes were studied by the panel as a common olfactory marker of hops but were not detected in Corsican wild hops, nor was 3-mercaptohexanol (a usual marker of this note) among the constituents of essential oils (Table A1). The blind olfactive analyzes of essential oils were triplicated, then all the scores from 0 to 5 for each accession were averaged and represented into aroma wheels.

For readability purposes, stations were grouped by type of aromatic profile according to their scores for the five studied scents. These profiles corroborate the estimated fragrance notes from chemical composition and the literature, presented in Table 4. Here again, three groups are formed (Figure 4): spicy and herbal and woody notes; medium notes with an equilibrated ratio between all scents except “tropical fruit”; and herbal and citrus notes. These results confirm the relation between the chemical composition and the olfactive profiles of Corsican hops, which both appear to be influenced by pedoclimatic conditions, altitude, and distance to the seashore.

**Figure 5:**
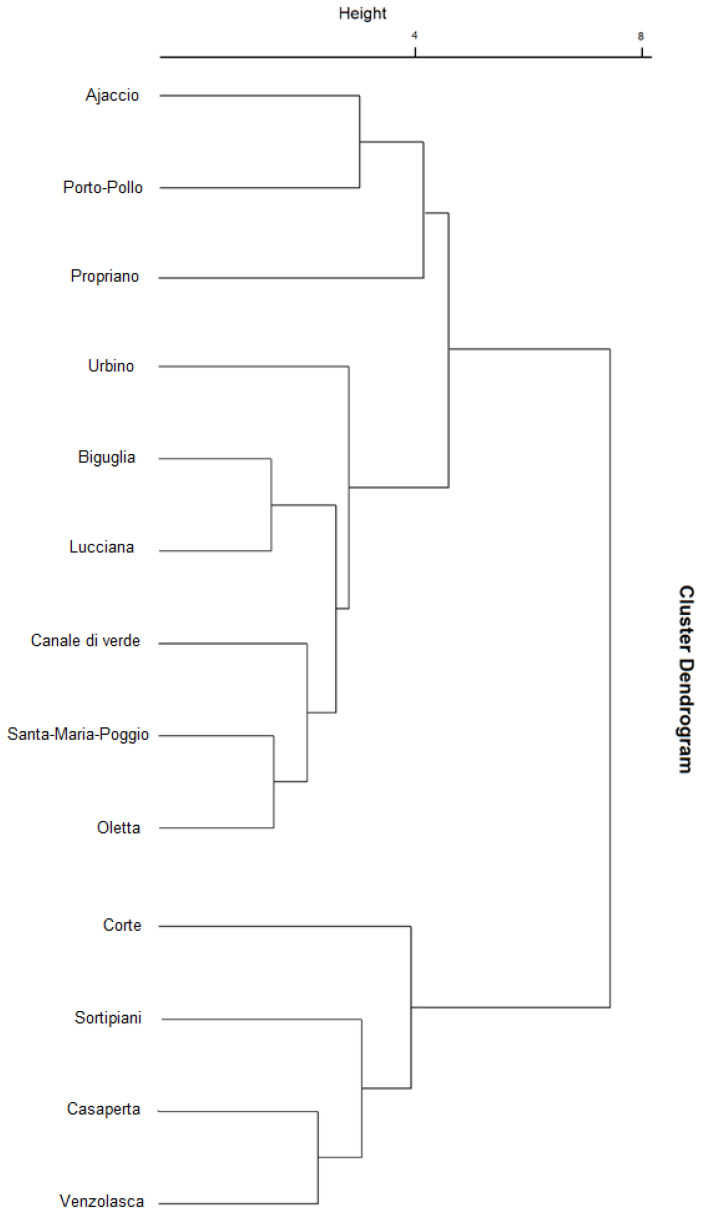
Dendrogram of the EOs from 13 harvesting stations.

To illustrate more clearly the similarities and dissimilarities between these stations and correlate them to environmental parameters, another statistical analysis was performed on the essential oils’ chemical composition using a dissimilarity matrix (Euclidean distance) and depicted as a dendrogram (Figure 5).

In Figure 5, two groups of samples (essential oils) share 78% of similarity. The first group is composed of the four accessions of *Corte, Sortipiani, Casaperta*, and *Venzolasca*, all of them located at medium altitude (80–400 m) inside the land. The second group is composed of the other accessions, all of them growing at low altitude levels (0–30 m) near the coastline. This observation could demonstrate the influence of altitude and possibly of salinity on the EOs profiles from Corsican wild hops. Within this group, a second differentiation emerges, with 54% of dissimilarity between *Ajaccio, Porto-Pollo*, and *Propriano*, all three located in the south–west part of the island on granitic soils, and the other accessions located in the north–east part of the island on sedimentary soils (Table 1). This observation could also highlight the influence of pedology on the EOs from Corsican wild hops.

### 3.4. Statistical Analysis—All Environmental Factors

To comprehend the compounds that are influenced by environmental factors, two-way ANOVAs were carried out between the seven discriminant compounds described earlier and parameters such as year, proximity to the sea, and pedology. The corresponding *p*-values are shown in Table 5.

In Table 5, the influence of the year of growth is only considered significant (*p*-value < 0.05) on the (*E*)-β-farnesene abundance. This could mean that the annual variability, probably induced by seasonal climatic conditions, can influence its concentration in hops volatile fraction and, by extension, the expression of a herbal–citrus aroma profile. This could mean that, for a same cultivar, the herbal–citrus predominance in a Corsican hopped beer might vary depending on the year of harvest. It is important to note that hops follow a strict annual cycle, with a vegetative state during winter season. Therefore, cones produced every year might be greatly influenced by seasonal changes observed at such small temporal scale. Future work must focus on the study of correlation between annual climatic data (such as pluviometry, mean temperature, or drought extent) and chemical composition of Corsican wild hops essential oils. Sampling over a longer period could also be considered.

The influence of proximity to the sea is significant for all compounds (especially α -selinene) except (*E*)-β-farnesene and zingiberene (*p*-values = 0.408 and 0.106, respectively). This result is unexpected regarding the high amounts of zingiberene detected in hops growing in altitude and relatively far from the coast (e.g., *Corte*). Therefore, altitude and salinity might not be the factors influencing the chemical particularity of these hops. On the other hand, the pedology factor is highly significant for all compounds (including zingiberene) except α-humulene and myrcene. *Corte* is a location with quite unique soil (schistous/granitic) and the great amounts of zingiberene could actually be dependent on pedology.

It was previously proven that the aroma profiles of Corsican hops’ essential oils are influenced by their chemical composition. Table 6 summarizes the scents that are believed to be influenced by the studied environmental factors.

The ANOVAs referred to in Table 5, as well as the other statistical analyzes presented in previous sections of this work, tend to prove the impact of environmental factors on the chemical composition of Corsican wild hops and, by extension, on their aroma profile.

### 3.5. Valorization in Food Industry

The chemical and sensory analyzes of the essential oils of Corsican hops have been of great importance in determining their brewing potential. Each growing station and their associated pedoclimatic conditions seem to exert an influence on the aroma profile. The abundance of zingiberene is remarkable. This compound, as well as its particularly desirable spicy profile, is found to a lesser extent in most commercial cultivars. Thus, commercially brewed Corsican hops could lead to the emergence of a highly typical organoleptic *bouquet* singular to Corsican beers and induced by these high zingiberene concentrations. In addition, their harvest would allow breweries outside Corsica to obtain ginger–spicy beers.

The impact of *terroir* on the aroma of Corsican hops has been described in this study. However, the brewing process is known to thermally and microbiologically alter certain organoleptic compounds [24], and further studies are needed to confirm these possible impacts on the aroma of beers. In association with local breweries, future prospections could be conceptualized on beer samples made with the thirteen different accessions used in this article. A new panel could then be formed and trained for sensory analysis of beverages. It is important to specify that the correlation between the aromatic profile of Italian hops and that of the beers brewed with them is already known in the food industry [15].

The industrial development of the 20th century led to a steady decline in the use of natural raw materials, particularly in the world of chemicals, pharmaceuticals, and the food industry. As climate change and the availability of resources become more problematic, to limit their environmental impact, today’s consumers tend to rethink their consumption modes patterns through local economy and the use of proximity products. The brewing world is no exception to this remodeling. By depicting their aroma profile, this article might enable Corsican breweries to use local wild hops as raw material. Following this work, we created the very first Corsican hop farm near the north coastline of the island. More than 2000 rhizomes were planted in the immediate vicinity of a brewery, which made it possible to study the impact of Corsican *terroir* on the cultivation of wild hops and foreign cultivars [11].

## 4. Conclusions

The aim of this study was to determine precisely and for the first time the variability of essential oils and the aroma of Corsican hops through both chemical and sensory analyzes. First, a sampling strategy on the scale of the island and the construction of the first hop farm in Corsica were initiated, with the intention of highlighting the typicality of Corsican hops and the impact of Corsican *terroir* on these different aspects.

Chemical and statistical analysis reveal a volatile profile that is quantitatively different from that of Italian hops, but also significant geographical and time-related diversity within Corsican hops where the main Eos’ compounds are β-selinene, zingiberene, myrcene, and α-selinene. The particularity of Corsican wild hops’ essential oils has been highlighted as well as their great interest due to the abundance of zingiberene, a compound with a ginger–spicy aroma highly valued in the brewing industry.

Beyond the substantial academic content, these advances allow the immediate economic and cultural enhancement of this local resource and could make Corsica the springboard for a Mediterranean brewing industry based on a guarantee of organoleptic typicality. Furthermore, this investigation demonstrates the importance of the hops cultivation’s parameters for producing beers with the desired organoleptic notes.

## Figures and Tables

**Table 1:** Pedology of the thirteen harvesting locations.

Location	Altitude (m)	Pedology	Humidity
*Corte*	400	Schistous/granitic	Strong
*Santa-Maria-Poggio*	30	Sedimentary	Average
*Oletta*	0	Sedimentary/schistous	Weak
*Ajaccio*	0	Granitic	Weak
*Biguglia*	0	Sedimentary	Average
*Lucciana*	0	Sedimentary	Average
*Venzolasca*	150	Sedimentary	Weak
*Canale di Verde*	0	Sedimentary	Average
*Casaperta*	80	Sedimentary/schistous	Weak
*Sortipiani*	130	Sedimentary/schistous	Weak
*Urbino*	0	Sedimentary	Average
*Porto-Pollo*	0	Granitic	Weak
*Propriano*	0	Granitic	Weak

**Table 2:** Nomenclature of the thirty-nine samples.

	Harvesting Years		
Stations	2019	2020	2021
*Corte*	1	2	3
*Canale di Verde*	4	5	6
*Santa-Maria-Poggio*	7	8	9
*Oletta*	10	11	12
*Ajaccio*	13	14	15
*Casaperta*	16	17	18
*Sortipiani*	19	20	21
*Porto-Pollo*	22	23	24
*Propriano*	25	26	27
*Biguglia*	28	29	30
*Urbino*	31	32	33
*Lucciana*	34	35	36
*Venzolasca*	37	38	39

**Table 3:** Quantitative variability of seven volatile compounds in Corsican hops (% of EO).

		Myrcene	Caryophyllene	Farnesene	Humulene	β-Selinene	Zingiberene	α-Selinene
Corsica	SD	9.2	6.8	8.2	9.8	6.5	6.5	8.1
	Average	11.1	11.7	8.3	12.2	6.8	6.9	6.7
Italy	SD	5.3	3.6	4.0	10.3	7.6	ND	4.7
	Average	20.5	10.6	3.6	17.1	6.9	ND	9.5

SD: standard deviation; ND: none detected.

**Table 4:** Corsican wild hops flavor notes estimated from their volatile profile and the literature.

Location	Flavor Impacting Compounds	Estimated Flavor Notes
*Corte*	Zingiberene; β-selinene; α-selinene	Spicy; herbal; woody
*Venzolasca*	Zingiberene; β-selinene; α-selinene	Spicy; herbal; woody
*Sortipiani*	Zingiberene; β-selinene; α-selinene	Spicy; herbal; woody
*Oletta*	Equally impacted	Medium notes
*Ajaccio*	Equally impacted	Medium notes
*Biguglia*	Equally impacted	Medium notes
*Porto-Pollo*	Equally impacted	Medium notes
*Propriano*	Equally impacted	Medium notes
*Santa-Maria-Poggio*	Myrcene	Herbal–citrus
*Lucciana*	(*E*)-β-farnesene	Herbal–citrus
*Canale di Verde*	(*E*)-β-farnesene	Herbal–citrus
*Casaperta*	myrcene	Herbal–citrus
*Urbino*	(*E*)-β-farnesene	Herbal–citrus

**Table 5:** Two-way ANOVAs between the seven discriminant compounds and environmental factors.

Compound	*p*-Values		
	Year	Proximity to the Sea	Pedology
Myrcene	0.929	**6.55 × 10^−3^**	0.089
(*E*)-β-caryophyllene	0.679	**5.68 × 10^−4^**	**0.011**
(*E*)-β-farnesene	**5.20 × 10^−3^**	0.408	**9.72 × 10^−4^**
α-humulene	0.688	**1.99 × 10^−3^**	0.458
β-selinene	0.226	**4.72 × 10^−5^**	**1.52 × 10^−6^**
Zingiberene	0.699	0.106	**2.01 × 10^−3^**
α-selinene	0.383	**5.44 × 10^−6^**	**0.028**

**Table 6:** Environmental factors influencing the aroma profile of Corsican hops.

Scents	Year	Proximity to the Sea	Pedology
Spicy (cloves)		X	X
Spicy (ginger)			X
Woody		X	X
Herbal	X	X	X
Citrus	X	X	X

## Data Availability

The data presented in this study are available on request from the corresponding author.

## Appendix A

**Table A1:** Chemical characterization of essential oils by GC–MS (% of essential oil).

**No**	**C1**				**C2**				**C3**				**C4**	**C5**	**C6**	**C7**				**C8**				**C9**				**C10**	**C11**	**C12**	**C13**				**C14**				**C15**				**C16**	**C17**	**C18**	**C19**				**C20**				**C21**				**C22**	**C23**	**C24**	**C25**				**C26**				**C27**			
1	0.1				1.7				0.1				0.1	0.0	0.1	0.1				0.1				10.8				0.2	0.2	2.2	2.4				0.5				0.0				4.5	15.7	15.6	0.0				20.9				0.0				0.5	0.0	0.0	0.1				0.1				0.2			
2	0.1				7.9				0.1				0.1	1.0	0.1	0.0				0.1				9.0				0.2	0.1	3.6	2.0				4.3				0.0				0.0	26.6	20.7	0.0				10.5				0.0				0.4	0.0	1.3	0.1				0.1				0.4			
3	0.0				0.7				0.0				0.0	0.2	2.0	0.0				0.2				11.8				0.2	0.0	2.5	0.9				3.7				0.0				0.0	23.4	0.0	0.0				23.6				0.0				0.4	0.0	1.0	0.1				0.1				1.2			
4	0.4				21.6				0.4				0.1	0.0	0.1	0.7				0.1				4.1				0.2	0.6	3.2	17.7				4.7				0.0				1.3	0.5	0.3	0.7				0.9				0.4				0.4	0.1	0.2	0.0				0.3				0.7			
5	0.0				1.0				0.0				0.1	0.0	0.7	0.1				0.1				11.7				0.5	0.2	13.8	26.6				0.1				1.8				0.2	1.6	1.6	2.8				2.3				0.1				1.1	2.3	0.2	1.5				1.0				0.1			
6	0.1				0.0				0.0				0.0	0.0	0.8	0.1				5.3				0.3				0.2	0.1	20.4	0.1				0.2				1.5				0.1	1.5	2.9	0.3				1.0				0.1				1.1	1.3	0.1	2.1				0.2				0.1			
7	0.2				4.1				0.2				0.0	0.0	0.9	0.0				1.1				2.7				0.4	0.1	1.7	11.7				2.3				1.8				7.7	3.6	0.2	0.0				1.2				0.4				0.2	0.1	0.2	0.0				0.7				0.0			
8	0.2				26.7				0.3				0.1	0.0	0.3	0.0				0.0				8.3				0.1	0.1	2.4	13.5				2.8				0.0				6.1	12.2	1.8	0.0				0.3				0.0				0.9	0.1	0.1	0.1				0.1				0.1			
9	0.0				5.4				0.1				0.3	0.0	0.7	0.0				0.0				16.7				0.5	0.0	26.6	17.8				0.1				0.4				1.7	1.8	1.4	0.9				1.1				0.1				1.6	0.2	3.1	0.3				0.0				0.3			
10	0.1				8.1				0.2				0.2	0.0	0.8	0.1				0.1				13.0				0.4	0.2	0.0	24.9				0.5				0.0				2.9	2.6	5.8	1.5				7.7				0.1				1.2	0.1	0.1	0.2				0.7				0.4			
11	0.1				0.1				0.0				0.0	0.0	0.3	0.1				0.0				11.3				1.4	0.0	2.7	15.5				2.3				0.0				1.4	12.3	5.3	1.5				2.3				0.1				1.5	0.1	0.0	0.1				0.1				0.1			
12	0.2				19.5				0.2				1.0	0.0	0.7	0.0				0.1				12.0				0.7	0.0	20.0	16.8				0.1				0.3				1.3	1.6	2.0	0.9				1.2				0.1				2.3	0.1	0.2	0.7				0.4				0.4			
13	0.3				20.1				0.1				0.4	0.0	0.3	0.0				0.2				5.2				1.1	1.4	0.0	18.3				2.0				1.7				0.2	6.7	13.7	3.1				1.3				0.3				2.1	0.1	0.1	1.9				1.8				0.3			
14	0.2				19.4				0.1				0.3	0.0	0.3	0.0				0.2				5.0				1.3	1.7	0.0	18.0				0.3				3.3				0.2	7.0	9.6	6.4				1.3				0.4				1.8	0.0	0.1	1.9				1.9				0.1			
15	0.2				18.2				0.0				0.3	0.0	0.1	0.0				4.9				1.1				1.3	0.0	0.0	15.7				2.3				0.2				0.3	6.4	16.2	4.6				1.2				0.3				0.0	0.2	1.9	2.3				2.0				0.2			
16	0.2				13.9				0.3				0.3	0.0	0.7	0.0				0.1				12.2				0.3	0.2	17.1	16.6				0.2				1.8				1.6	4.1	8.1	0.1				0.9				0.0				1.5	0.1	0.1	0.1				0.0				0.1			
17	0.2				12.7				0.3				0.3	0.1	0.4	0.0				0.1				13.0				0.4	0.2	18.2	15.8				0.3				1.9				1.4	4.8	7.7	0.1				0.9				0.1				1.7	0.1	0.2	0.2				0.1				0.1			
18	0.0				10.6				0.1				0.0	0.5	0.0	0.0				0.0				13.8				0.0	0.0	25.1	15.1				0.4				1.3				0.9	0.6	1.1	0.4				0.1				0.0				1.3	2.5	0.1	0.2				0.3				0.1			
19	0.1				7.4				0.1				0.0	0.3	0.0	0.0				0.0				16.2				0.3	0.5	1.0	2.9				0.4				3.2				0.0	5.0	13.1	0.4				18.0				0.1				0.9	0.7	0.1	3.0				3.1				0.1			
20	0.1				5.1				0.1				0.0	0.7	0.1	0.0				0.0				17.1				0.6	0.3	1.5	2.8				0.5				3.7				0.0	5.3	12.4	0.4				18.1				0.1				0.9	0.6	0.1	3.4				3.4				0.1			
21	0.1				4.5				0.1				0.0	0.9	0.1	0.0				0.0				19.5				0.6	0.4	2.7	2.4				0.4				3.7				0.0	4.1	9.5	0.4				15.9				0.1				1.2	0.7	0.1	3.5				3.4				0.1			
22	0.3				24.5				0.1				0.2	0.0	0.7	0.0				2.1				4.1				0.3	0.1	4.1	1.6				0.1				1.2				3.9	8.0	22.1	2.0				0.5				0.3				2.1	0.0	1.1	2.0				1.8				0.3			
23	0.2				28.3				0.1				0.2	0.0	0.4	0.0				0.1				5.8				0.4	0.0	3.4	0.1				0.2				1.7				4.1	8.8	17.8	3.1				0.8				0.4				2.9	0.1	0.2	2.0				2.0				0.1			
24	0.3				26.7				0.2				0.2	0.0	0.1	0.1				5.2				0.5				0.1	0.1	2.3	7.2				0.2				0.3				1.1	7.3	16.7	4.1				0.0				0.0				0.5	0.1	2.4	1.8				1.5				0.3			
25	0.1				5.2				0.1				0.5	0.0	0.5	0.0				0.0				16.2				0.4	0.0	0.6	29.1				0.3				2.1				1.9	6.0	2.0	0.9				9.3				0.9				0.4	1.9	0.5	0.0				0.0				0.1			
26	0.1				7.1				0.1				0.4	0.0	0.6	0.0				0.0				17.9				0.4	0.0	0.1	28.8				0.4				2.7				1.9	6.1	2.3	0.8				10.0				1.0				0.2	2.0	0.1	0.3				0.1				0.0			
27	0.2				15.2				0.2				1.0	0.0	0.5	0.0				0.0				17.9				0.2	0.1	6.7	20.2				0.4				0.0				2.1	1.5	7.1	0.4				9.2				0.1				0.1	0.1	1.7	0.3				0.1				0.2			
28	0.1				8.1				0.0				0.3	0.0	0.5	0.0				0.1				11.0				0.5	0.0	8.2	0.3				2.6				0.0				1.4	9.5	4.8	0.4				17.4				0.5				0.3	0.0	0.4	0.4				0.0				0.1			
29	0.0				0.9				0.0				0.1	0.0	0.2	0.0				0.1				16.6				0.5	0.0	9.1	0.5				2.4				0.0				1.1	10.4	4.7	0.4				16.4				0.2				0.4	0.0	0.5	0.1				0.0				0.1			
30	0.2				16.7				0.2				0.4	0.0	0.5	0.0				0.0				10.8				0.0	0.0	14.4	22.6				0.3				1.1				1.0	0.5	0.7	0.4				0.2				1.1				2.3	0.0	0.2	0.1				0.2				0.2			
31	0.1				18.1				0.3				0.0	0.6	0.9	0.0				0.0				10.2				0.3	0.0	15.6	26.7				0.3				1.2				0.9	1.9	0.4	0.7				2.0				0.0				0.4	3.1	0.0	0.0				0.0				0.0			
32	0.4				21.9				0.2				0.0	0.5	0.7	0.0				0.0				13.8				0.5	0.0	16.1	23.3				0.3				1.5				0.8	1.1	0.9	0.4				1.4				0.0				0.1	2.6	0.0	0.3				0.1				0.1			
33	0.0				0.7				0.0				0.1	0.0	0.7	0.0				0.1				15.2				0.5	0.0	18.4	25.4				0.1				0.3				1.8	3.0	3.3	0.9				1.6				0.0				0.2	2.9	0.1	0.1				0.3				0.1			
34	0.4				9.0				0.1				0.4	0.1	0.6	0.0				0.9				14.0				0.0	0.0	10.0	15.2				0.0				1.1				0.7	2.4	5.1	0.3				0.0				0.1				0.9	0.0	0.1	0.0				0.3				0.2			
35	0.1				14.3				0.0				0.1	0.1	0.4	0.0				0.1				10.3				0.3	0.5	16.7	13.5				0.3				2.1				1.3	3.9	7.5	0.3				0.1				0.3				1.5	0.1	0.1	0.1				0.1				0.1			
36	0.4				26.7				0.3				0.1	1.2	0.1	0.2				5.9				0.7				0.0	0.0	22.7	4.4				1.2				0.1				1.0	1.3	3.6	0.5				0.5				0.1				1.0	0.1	0.1	0.0				0.1				0.3			
37	0.1				0.1				0.0				0.0	0.0	0.3	0.2				0.1				30.1				0.2	0.0	3.5	0.2				0.3				3.8				0.0	17.2	1.1	0.0				21.9				0.0				0.0	0.1	0.2	0.2				0.0				0.0			
38	0.0				0.4				0.0				0.0	0.0	0.1	0.1				0.2				28.4				0.2	0.2	4.4	0.1				0.7				4.7				0.0	21.9	2.1	0.0				20.7				0.1				0.5	0.1	0.1	0.1				0.1				0.1			
39	0.0				0.6				0.0				0.0	0.0	0.5	0.0				0.0				17.9				0.0	0.0	2.3	0.8				4.1				0.0				0.0	5.5	17.0	0.7				20.1				0.5				0.1	1.1	0.1	0.1				0.1				0.4			
**No**	**C28**			**C29**			**C30**			**C31**			**C32**	**C33**	**C34**	**C35**			**C36**			**C37**			**C38**			**C39**	**C40**	**C41**	**C42**			**C43**			**C44**			**C45**			**C46**	**C47**	**C48**	**C49**			**C50**			**C51**			**C52**			**C53**	**C54**	**C55**	**C56**			**C57**			**C58**			**C59**		
1	0.1			0.1			0.0			1.2			0.3	0.4	2.6	0.9			0.1			0.0			0.1			0.1	0.1	0.1	2.5			0.1			0.0			0.1			0.1	0.1	0.0	0.0			0.0			0.1			0.1			0.0	3.7	0.1	0.1			0.4			0.0			1.1		
2	0.1			0.1			0.0			0.1			0.1	0.1	1.6	2.4			0.1			0.1			0.0			0.3	1.1	0.0	0.0			0.1			0.1			0.1			0.1	0.1	0.1	0.1			0.1			0.3			0.3			0.0	0.1	1.1	0.1			0.3			0.8			0.1		
3	0.1			0.2			0.0			0.1			0.1	0.2	2.8	3.1			0.1			0.0			0.0			0.2	0.1	0.0	0.7			0.0			0.1			0.0			0.0	0.0	0.0	0.0			0.0			0.2			0.1			0.1	2.5	1.1	0.1			0.3			0.0			3.9		
4	1.0			0.7			0.0			8.4			0.7	0.3	1.3	0.1			0.3			0.4			0.3			0.8	0.0	0.2	0.0			0.7			0.0			0.3			0.2	0.9	0.4	0.0			0.6			1.2			0.1			0.1	3.9	0.1	0.0			1.2			0.3			0.2		
5	0.1			0.1			0.2			1.8			0.1	0.4	3.9	1.1			0.2			0.0			0.3			2.6	0.1	0.0	0.1			0.4			0.0			0.8			0.0	0.1	0.2	0.1			0.0			0.5			0.0			0.1	0.2	0.6	0.0			0.3			0.1			4.1		
6	0.1			0.6			1.4			11.3			0.4	0.7	2.1	1.7			0.4			0.0			0.5			2.8	0.0	0.0	0.7			0.2			0.1			0.1			0.1	0.1	0.3	0.1			0.0			0.1			0.2			0.1	3.8	0.3	0.4			1.5			0.0			4.6		
7	0.0			0.8			0.4			16.6			1.2	0.4	1.3	0.1			0.4			0.2			0.0			0.0	0.9	0.0	0.9			0.0			0.0			0.0			0.0	0.3	0.2	0.0			0.0			1.0			0.1			0.1	0.1	0.4	0.1			0.3			0.0			0.3		
8	0.1			0.2			0.0			0.0			0.8	0.1	3.8	2.6			0.4			0.0			0.2			2.7	0.3	0.0	0.8			0.1			0.1			0.4			0.1	0.1	0.3	0.2			0.1			0.7			0.1			0.1	0.1	0.1	0.1			0.2			0.0			0.2		
9	0.0			0.3			0.2			0.9			0.3	0.0	1.7	1.9			0.0			0.0			0.3			3.0	0.0	0.0	0.1			0.2			0.0			0.4			0.0	0.1	0.4	0.1			0.0			0.1			0.1			0.1	0.0	0.2	0.0			0.2			0.0			0.7		
10	0.5			0.2			2.7			2.6			0.3	0.4	2.2	1.1			0.3			0.0			0.0			0.8	0.1	0.2	0.0			0.7			0.1			0.3			0.0	0.4	0.6	0.3			0.2			0.8			0.1			0.1	1.2	0.5	0.1			0.2			1.5			0.2		
11	0.1			0.4			0.5			0.4			0.1	0.1	2.5	0.8			0.2			0.1			0.1			0.1	0.1	0.1	0.0			0.0			0.0			0.2			0.0	0.0	0.0	0.0			0.0			0.1			0.0			0.0	0.0	0.0	0.0			0.0			0.0			3.4		
12	0.0			0.9			0.2			0.4			0.3	0.4	1.1	0.0			0.0			0.0			0.6			1.5	0.1	0.2	0.0			0.6			0.0			0.3			0.0	0.2	0.5	0.1			0.1			0.3			0.0			0.0	0.3	0.2	0.3			0.2			0.0			0.5		
13	0.1			0.1			0.0			0.5			0.3	0.3	0.7	0.3			0.6			0.4			0.3			1.1	0.1	0.4	0.1			0.2			0.1			0.2			0.0	0.7	0.3	0.0			0.0			0.6			0.1			0.1	0.1	0.1	0.5			0.2			0.0			0.2		
14	0.2			0.1			0.0			0.4			0.3	0.2	0.8	0.5			0.5			0.0			0.0			0.8	0.1	0.2	0.2			0.2			0.1			0.1			0.0	0.4	0.2	0.3			0.0			0.6			0.1			0.1	0.5	0.6	0.2			1.5			0.0			0.0		
15	0.3			0.4			0.0			0.1			0.5	0.3	0.9	0.0			0.5			0.2			0.0			1.1	0.2	0.0	0.4			0.2			0.0			0.1			0.3	0.1	0.0	0.2			0.1			0.4			0.0			0.0	0.1	0.4	0.1			0.2			0.0			0.8		
16	0.0			0.2			0.3			0.5			0.3	0.5	1.7	0.8			0.3			0.0			0.3			2.8	0.1	0.0	0.4			0.1			0.0			0.2			0.2	0.1	0.4	0.1			0.1			0.4			0.1			0.1	0.1	0.2	0.1			0.1			0.0			0.9		
17	0.0			0.1			0.3			0.4			0.2	0.4	1.9	1.1			0.2			0.0			0.3			2.9	0.1	0.0	0.1			0.1			0.0			0.3			0.1	0.1	0.5	0.2			0.1			0.5			0.1			0.1	0.1	0.1	0.1			0.1			0.0			0.3		
18	0.0			0.5			0.2			0.7			0.0	0.3	0.9	1.4			0.3			0.0			0.4			1.4	0.0	0.0	0.7			0.3			0.0			0.4			0.0	0.1	0.4	0.0			0.0			0.1			0.0			0.0	0.2	0.0	0.2			0.3			0.0			1.4		
19	0.0			0.1			0.1			0.0			0.3	0.2	3.5	2.9			0.7			0.0			0.3			1.3	0.1	0.0	1.5			0.0			0.0			0.0			0.0	0.1	0.4	0.0			0.0			0.4			0.0			0.0	0.2	0.4	0.4			0.6			0.0			0.1		
20	0.0			0.2			0.1			0.1			0.4	0.4	3.6	3.4			0.9			0.0			0.4			1.4	0.1	0.0	1.5			0.1			0.0			0.2			0.0	0.1	0.3	0.0			0.0			0.2			0.0			0.0	0.2	0.8	0.1			0.4			0.0			0.1		
21	0.0			0.1			0.1			0.1			0.4	0.7	2.9	3.8			0.9			0.0			0.4			0.8	0.1	0.0	2.0			0.1			0.0			0.2			0.0	0.1	0.3	0.0			0.0			0.3			0.0			0.0	0.1	0.7	0.3			0.9			0.0			0.4		
22	0.4			1.2			0.1			0.2			0.1	0.2	2.1	0.6			0.3			0.0			0.6			1.5	0.0	0.0	0.3			0.3			0.0			0.2			0.0	0.1	0.4	0.1			0.0			0.1			0.2			0.0	0.1	0.4	0.1			0.1			0.0			0.8		
23	0.1			1.4			0.0			0.3			0.2	0.2	2.6	0.7			0.8			0.0			0.4			1.3	0.0	0.0	0.2			0.4			0.0			0.3			0.0	0.2	0.3	0.1			0.1			0.4			0.1			0.0	0.2	0.3	0.2			0.0			0.0			0.1		
24	0.0			0.0			0.0			0.1			0.2	0.3	0.9	2.4			0.3			0.0			0.5			1.8	0.0	0.0	0.7			0.6			0.0			0.3			0.0	0.5	0.6	0.2			0.1			0.0			0.0			0.0	0.0	0.4	0.1			0.3			0.0			1.0		
25	0.0			0.0			0.8			0.5			0.1	0.2	3.8	0.0			0.1			0.1			0.4			1.4	0.1	0.0	1.3			0.1			0.0			0.1			0.0	0.2	0.2	0.3			0.1			0.5			0.1			0.0	0.1	0.0	0.1			0.1			0.0			0.6		
26	0.0			0.4			1.1			0.4			0.2	0.2	4.0	0.0			0.0			0.0			0.3			1.5	0.1	0.0	1.0			0.2			0.0			0.2			0.0	0.1	0.2	0.1			0.1			0.4			0.1			0.0	0.2	0.0	0.3			0.0			0.0			0.0		
27	0.0			0.0			0.3			0.3			0.1	0.2	3.5	0.0			0.2			0.7			0.6			1.8	0.0	0.0	0.8			0.2			0.0			0.3			0.0	0.3	0.0	0.1			0.1			0.5			0.1			0.0	0.2	0.4	0.2			0.4			0.0			0.7		
28	0.0			0.1			0.5			0.1			0.1	0.0	2.4	1.5			0.0			0.0			0.0			1.1	0.0	0.0	0.6			0.0			0.0			0.3			0.1	0.0	0.2	0.0			0.0			0.0			0.0			0.0	0.1	0.6	0.2			0.8			0.7			9.9		
29	0.0			0.1			0.1			0.4			0.1	0.0	1.9	1.3			0.1			0.0			0.0			0.8	0.0	0.0	0.1			0.0			0.0			0.1			0.1	0.0	0.1	0.0			0.0			0.1			0.0			0.0	0.1	0.4	0.1			0.1			0.6			9.7		
30	0.0			0.1			0.4			0.4			0.0	0.2	0.7	0.8			0.2			0.4			0.2			2.0	0.5	0.0	1.2			0.0			0.0			0.2			0.0	0.2	0.3	0.2			0.1			0.0			0.1			0.1	0.1	0.2	0.2			0.6			0.0			1.2		
31	0.0			0.2			0.4			0.2			0.2	0.2	1.5	0.9			0.0			0.1			0.2			1.4	0.0	0.0	0.9			0.3			0.0			0.5			0.7	0.0	0.2	0.1			0.1			0.4			0.0			0.0	0.5	0.0	0.5			0.4			0.0			0.5		
32	0.0			0.1			0.6			0.1			0.1	0.2	1.6	0.1			0.0			0.1			0.3			1.5	0.0	0.0	1.1			0.2			0.0			0.4			0.5	0.3	0.4	0.1			0.1			0.3			0.1			0.0	0.2	0.0	0.2			0.1			0.0			0.0		
33	0.0			0.1			1.8			0.2			0.2	0.0	1.9	1.8			0.4			0.0			0.3			1.6	0.0	0.1	1.8			0.2			0.1			0.4			0.1	0.0	0.2	0.1			0.1			0.2			0.1			0.1	0.7	0.0	0.5			0.5			0.0			1.7		
34	0.2			0.1			3.1			0.4			0.2	0.1	3.1	0.4			0.0			0.0			0.1			0.2	0.1	0.1	0.1			0.2			0.0			0.2			0.1	0.1	0.2	0.1			0.0			0.3			0.3			0.2	1.1	0.0	0.0			0.5			0.0			6.8		
35	0.1			0.2			4.0			0.5			0.5	0.2	2.2	1.1			0.3			0.0			0.1			0.5	0.1	0.1	0.1			0.7			0.0			0.3			0.1	0.0	0.2	0.1			0.0			0.4			0.1			0.1	0.1	0.0	0.0			0.0			0.0			3.5		
36	0.1			0.2			0.3			0.2			0.1	0.2	1.3	1.8			0.2			0.7			0.2			2.1	0.0	0.1	0.1			0.5			0.0			0.1			0.0	0.4	0.1	0.6			0.1			1.8			0.3			0.4	0.1	0.0	0.1			0.1			0.0			1.8		
37	0.1			0.2			0.0			0.2			0.1	0.2	1.5	0.6			0.1			0.0			0.1			0.1	0.3	0.0	0.3			0.1			0.0			0.4			0.0	0.0	0.1	0.2			0.3			0.1			0.2			0.3	0.9	0.3	0.1			0.4			2.3			3.9		
38	0.1			0.1			0.1			1.3			0.1	0.4	2.2	0.4			0.1			0.0			0.1			0.3	0.5	0.0	0.1			0.1			0.0			0.1			0.0	0.0	0.0	0.1			0.1			0.4			0.1			0.1	1.2	0.0	0.1			0.1			1.9			2.8		
39	0.0			3.4			0.1			1.6			0.2	0.2	3.3	3.4			0.1			0.0			0.2			0.9	0.1	0.0	0.5			0.1			0.0			0.1			0.0	0.0	0.1	0.2			0.1			0.7			0.0			0.1	0.0	0.1	0.1			0.4			0.0			4.8		

**Table A2:** Determination key of the samples analyzed by GC–MS.

Id	Compounds	RI litt ^a^	RI a ^b^	RI p ^c^	Range (% of EO)	Averages	Standard Deviations
	**Hydrocarbons**						
C1	β-Pinene	964	970	1110	*tr*—0.4	0.1	0.1
*C2*	*Myrcene*	*981*	*976*	*1159*	*tr—28.3*	*11.1*	*9.2*
C3	Limonene	1029	1020	1199	*tr*—0.4	0.1	0.1
C4	(*Z*) β-Ocimene	1039	1024	1230	*tr*—1.0	0.2	0.1
C5	(*E*) β-Ocimene	1050	1034	1247	*tr*—1.2	0.2	0.1
C6	α-Ylangene	1373	1375	1476	*tr*—2.0	0.5	0.3
C7	α-Copaene	1381	1379	1488	*tr*—0.7	0.0	0.1
C8	Isocaryophyllene	1402	1407	1571	*tr*—5.9	0.7	0.4
*C9*	*(E)-β-Caryophyllene*	*1414*	*1424*	*1591*	*0.3—30.1*	*11.7*	*6.8*
C10	β-Copaene	1432	1431	1581	*tr*—1.4	0.4	0.2
C11	(*E*)-α-Bergamotene	1432	1432	1580	*tr*—1.7	0.2	0.1
*C12*	*(E)-β-Farnesene*	*1445*	*1448*	*1661*	*tr—26.6*	*8.3*	*8.2*
*C13*	*α-Humulene*	*1455*	*1456*	*1665*	*0.1—29.1*	*12.2*	*9.8*
C14	4,5-Di-*epi*-aristolochene	1469	1467	1665	*tr*—4.7	1.1	0.8
C15	γ-Muurolene	1477	1471	1681	*tr*—4.7	1.3	0.7
C16	γ-Himachalene	1479	1479	1693	*tr* –7.7	1.5	0.8
*C17*	*β-Selinene*	*1485*	*1483*	*1712*	*0.5—26.6*	*6.8*	*6.5*
*C18*	*Zingiberene*	*1493*	*1489*	*1717*	*tr—22.1*	*6.9*	*6.5*
C19	Valencene	1495	1497	1719	*tr*—6.4	1.0	0.5
*C20*	*α-Selinene*	*1505*	*1494*		*tr—23.6*	*6.7*	*8.1*
C21	(*E,E*) α-Farnesene	1506	1498	1744	*tr*—1.1	0.2	0.3
C22	γ-Cadinene	1513	1507	1752	*tr*—2.9	0.9	0.5
C23	Calamenene	1522			*tr*—3.1	0.6	0.4
C24	δ-Cadinene	1524	1516	1752	*tr*—3.1	0.4	0.3
C25	γ-Bisabolene	1529			*tr*—3.5	0.8	0.3
C26	α-Cadinene	1538	1535	1743	*tr*—3.4	0.7	0.4
	**Alcohols**						
C27	Linalool	1087	1081	1544	*tr*—1.2	0.2	0.2
C28	α-Terpineol	1179	1179	1700	*tr*—1.0	0.1	0.2
C29	8-Caryolanol	1562	1559	2044	*tr*—3.4	0.4	0.3
C30	Viridiflorol	1593	1591	2089	*tr*—4.0	0.5	0.3
C31	Humulol	1601	1588	2165	*tr*—16.6	1.4	3.3
C32	Zingiberenol 1	1614	1599	2109	*tr*—1.2	0.3	0.2
C33	Zingiberenol 2	1620	1613	2190	*tr*—0.7	0.3	0.2
C34	α-Cadinol	1642	1641	2231	0.7—4.0	2.2	1.0
C35	4α-Eudesm-11-enol	1651	1642	2241	*tr*—3.8	1.2	0.5
C36	α-Bisabolol	1680	1672	2217	*tr*—0.9	0.3	0.2
	**Ketones**						
C37	2-Nonanone	1091	1070	1388	*tr*—0.7	0.1	0.2
C38	2-Decanone	1172	1176	1495	*tr*—0.6	0.2	0.2
C39	2-Undecanone	1291	1273	1592	*tr*—3.0	1.3	0.7
C40	2-Dodecanone	1371	1385	1711	*tr*—1.1	0.1	0.2
C41	2-Tetradecanone	1576	1580	1909	*tr*—0.4	0.0	0.1
C42	(*Z*) 2-Pentadec-6-enone	1652	1647		*tr*—2.5	0.6	0.4
	**Aldehydes**						
C43	Nonanal	1084	1083	1394	*tr*—0.7	0.2	0.1
C44	Decanal	1184	1185	1498	*tr*—0.1	0.0	0.0
C45	Geranial	1242	1244	1731	*tr*—0.8	0.2	0.2
	**Esters**						
C46	2-methylbutyl isobutyrate	989	1004	1176	*tr*—0.7	0.1	0.1
C47	Methyl heptanoate	1006	1010		*tr*—0.9	0.2	0.2
C48	Methyl 6-methylheptanoate	1070	1068	1338	*tr*—0.6	0.2	0.1
C49	Methyl octanoate	1110	1110	1396	*tr*—0.6	0.1	0.1
C50	Methyl nonanoate	1210	1205		*tr*—0.6	0.1	0.1
C51	Methyl 4-decenoate	1290	1290	1611	*tr*—1.8	0.4	0.3
C52	Methyl geraniate	1301	1301	1680	*tr*—0.3	0.1	0.1
C53	Methyl decanoate	1307	1307	1581	*tr*—0.4	0.1	0.1
	**Others**						
C54	Caryophyllene oxide	1571	1576	1980	*tr*—3.9	0.6	0.4
C55	Humulene epoxide I	1593	1593	2040	*tr*—1.1	0.3	0.2
C56	Humulene epoxide II	1602	1601	2044	*tr*—0.5	0.2	0.1
C57	Humulene epoxide III	1626	1626		*tr*—1.5	0.4	0.3
C58	Aromadendrene oxide	1650	1617	2002	*tr*—2.3	0.2	0.2
C59	Hexadecanoic acid	1961			*tr*—9.9	1.9	1.2

^a^: Literature non-polar retention indices [25]; ^b^: experimental non-polar indices; ^c^: experimental polar indices; italic: compounds showing significant variations.
